# Systems Biology–Derived Genetic Signatures of Mastitis in Dairy Cattle: A New Avenue for Drug Repurposing

**DOI:** 10.3390/ani12010029

**Published:** 2021-12-23

**Authors:** Somayeh Sharifi, Maryam Lotfi Shahreza, Abbas Pakdel, James M. Reecy, Nasser Ghadiri, Hadi Atashi, Mahmood Motamedi, Esmaeil Ebrahimie

**Affiliations:** 1 Department of Animal Sciences, College of Agriculture, Isfahan University of Technology, Isfahan 84156-83111, Iran; pakdel@cc.iut.ac.ir; 2 Department of Animal Science, Iowa State University, Ames, IA 50011, USA; jreecy@iastate.edu; 3 Department of Computer Engineering, Shahreza Campus, University of Isfahan, Isfahan 86149-56841, Iran; m.lotfi@shr.ui.ac.ir; 4 Department of Electrical and Computer Engineering, Isfahan University of Technology, Isfahan 84156-83111, Iran; nghadiri@iut.ac.ir; 5 Department of Animal Science, Shiraz University, Shiraz 71946-84334, Iran; hadiatashi@gmail.com; 6 Department of Animal Sciences, University of Tehran, Tehran 1417935840, Iran; motamedim7@gmail.com; 7 Genomics Research Platform, School of Life Sciences, College of Science, Health and Engineering, La Trobe University, Melbourne, VIC 3086, Australia; 8 School of Animal and Veterinary Sciences, The University of Adelaide, Adelaide, SA 5371, Australia; 9 School of BioSciences, The University of Melbourne, Melbourne, VIC 3010, Australia

**Keywords:** drug repositioning, drug targets, *E. coli*, mastitis, gene regulation, inflammation

## Abstract

**Simple Summary:**

Therapeutic success of bovine mastitis depends mainly on accurately diagnosing the type of pathogen involved. Despite the development prospects for bovine mastitis diagnosis, including new biomarker discovery to target specific pathogens with high sensitivity and specificity, treatment studies have shown controversial results, and the most efficient, safe, and economical treatments for mastitis are still topics of scientific debate. The goal of this research is the integration of different levels of systems biology data to predict candidate drugs for the control and management of *E. coli* mastitis. We propose that the novel drugs could be used by pharmaceutical scientists or veterinarians to find commercially efficacious medicines.

**Abstract:**

Mastitis, a disease with high incidence worldwide, is the most prevalent and costly disease in the dairy industry. Gram-negative bacteria such as *Escherichia coli* (*E. coli*) are assumed to be among the leading agents causing acute severe infection with clinical signs. *E. Coli*, environmental mastitis pathogens, are the primary etiological agents of bovine mastitis in well-managed dairy farms. Response to *E. Coli* infection has a complex pattern affected by genetic and environmental parameters. On the other hand, the efficacy of antibiotics and/or anti-inflammatory treatment in *E. coli* mastitis is still a topic of scientific debate, and studies on the treatment of clinical cases show conflicting results. Unraveling the bio-signature of mastitis in dairy cattle can open new avenues for drug repurposing. In the current research, a novel, semi-supervised heterogeneous label propagation algorithm named Heter-LP, which applies both local and global network features for data integration, was used to potentially identify novel therapeutic avenues for the treatment of *E. coli* mastitis. Online data repositories relevant to known diseases, drugs, and gene targets, along with other specialized biological information for *E. coli* mastitis, including critical genes with robust bio-signatures, drugs, and related disorders, were used as input data for analysis with the Heter-LP algorithm. Our research identified novel drugs such as Glibenclamide, Ipratropium, Salbutamol, and Carbidopa as possible therapeutics that could be used against *E. coli* mastitis. Predicted relationships can be used by pharmaceutical scientists or veterinarians to find commercially efficacious medicines or a combination of two or more active compounds to treat this infectious disease.

## 1. Introduction

Clinical mastitis, an ongoing problem for dairy producers, results in considerable economic losses and has led to an increased risk of culling and death in dairy cows [1,2,3]. Mastitis control programs targeting the prevalence of contagious mastitis pathogens have led to a reduction in the incidence of *Staphylococcus aureus* and *Streptococcus agalactiae* mastitis; as a result, environmental mastitis pathogens such as *Escherichia coli (E. coli)* have become the primary etiological agents of bovine mastitis on well-managed dairy farms [3,4,5,6]. *E. coli* infection can cause either subclinical infection of the mammary gland or severe systemic disease. Although intramammary *E. coli* infections with acute inflammation may be spontaneously eradicated by host defenses, in extreme cases, they can be fatal [3,7,8,9]. In addition, untreated infections are often associated with significant economic damage due to the longer duration of infection, lower milk yield, and the potential for pathological changes to the mammary gland [3,10].

Successful therapeutic outcomes for bovine mastitis depend mainly on accurate diagnosis, the severity of udder pathology, drug selection, relevance of route of administration, supportive treatment, and elimination of predisposing factors. Accurate diagnosis of the kind of pathogen improves clinical and microbiological efficacy and helps prevent the emergence and spread of resistant microorganisms. Despite the prospects for bovine mastitis diagnosis, including new biomarker discovery with high sensitivity and specificity to specific pathogens [3,11,12,13,14], the most efficient, safe, and economical treatments for mastitis are still topics of scientific debate [3,15,16]. Coliform mastitis is an acute and potentially lethal type of bovine mastitis. The great majority of these coliform bacteria are *E. coli*. Because coliform mastitis can be so severe in its manifestation and consequences, the goal of therapy is to preserve the cow’s life and minimize harmful sequelae. Generally, narrow and/or broad-spectrum antimicrobial agents are used as the primary antimicrobial treatment for mastitis in dairy herds, specifically for infections caused by Gram-positive bacteria. For the problems associated with antibiotic therapy, including the emergence of antibiotic-resistant strains, and the concern about antibiotics entering the food chain, efforts are being made to substitute the customary strategies for new non-antimicrobial agents, including bacteriophages, vaccination, nanoparticles, cytokines, homeopathy, natural compounds from plants, animals, and bacteria, or the discovery of new drugs that are effective against mastitis pathogens [3,15,17]. In *E. coli* mastitis with mild to moderate clinical signs, non-antimicrobial approaches including glucocorticoids, nonsteroidal anti-inflammatory drugs (NSAIDs), frequent milking, fluid therapy, and lactoferrin have been suggested as alternatives to antimicrobials [18] to preserve milk production, alleviate clinical signs, and reduce mortality. In coliform mastitis, infection and, consequently, clinical signs, are mainly caused by lipopolysaccharide (LPS); thus, treatment should be targeted at those effects. In cases of severe *E. coli* mastitis, although treatment studies have shown controversial results, broad-spectrum antimicrobial agents such as fluoroquinolones [18], Cephalexin, Gentamicin, and Dexamethasone [19] are recommended due to the risk of unlimited growth of bacteria in the mammary gland and to avoid the risk of bacteremia. Evidence for the efficacy of intramammary-administered antimicrobial treatment for *E. coli* mastitis is limited [18]. 

Today, there is a large amount of available biological data, which is very useful for many applications. The integration of drug, disease, and gene target information, in addition to an understanding of the drugs’ effects and functions in the body, can help formulate strategies for drug repositioning (repurposing) and the possible identification of disease treatments.

In the current study, we integrated different levels of biological data (data relevant to diseases, drugs, and gene targets) using a novel, semi-supervised heterogeneous label propagation algorithm named Heter-LP, which applies both local and global network features for data integration to potentially identify novel therapeutic avenues for the treatment of *E. coli* mastitis.

Currently, the first step to drug development is the use of previously known drugs; this is known as drug repositioning. This approach has attracted a lot of interest in recent years because of the increased speed of the process, reduced drug safety concerns, and lower cost. Different computational tools for drug repositioning analysis and methods for the prediction of drug-target interactions have been presented in a recent review [20]. Among them, Heter-LP was selected because of advantages such as accuracy, lack of requirement for negative samples, ability to predict trivial and non-trivial relationships between drugs, diseases, and protein targets, and ability to use heterogeneous data [21,22].

## 2. Materials and Methods

In the current study, we used Heter-LP, a systems biology approach, to discover drugs to be repositioned for *E. coli* mastitis in the dairy cow by using different levels of biological data [22]. So far, the main focus has been on networks with the same kind of nodes and the same kind of edges, known as homogeneous networks. However, the most recently encountered problems need more details that could not be presented by a simple homogeneous network. It has been observed that the use of network-based methods in the integration of biological data at different levels has yielded good results. The utility of Heter-LP to discover new drug repositioning options for rare diseases in humans has been explored previously [21]. Heter-LP is a semi-supervised learning method based on label propagation on a heterogeneous network consisting of three types of nodes (targets, drugs, and diseases) and six different kinds of edges (three kinds of similarities and three kinds of associations) [22]. 

### 2.1. The Input Network Construction

For the constructed network, six separate matrices were prepared: (1) drug similarities, (2) disease similarities, (3) target similarities, (4) drug-disease relations, (5) disease-target relations, and (6) drug-target relations. 

Different essential data for each part were gathered and organized as a comprehensive dataset for a previous study (available through GitHub [https://github.com/MLotfiSH/Heter-LP, accessed on 26 March 2021] and the DKR site [http://dkr.iut.ac.ir/projects, accessed on 28 March 2021]) [21]. The data resources are summarized in Table 1. For example, three different criteria used to construct the drug similarities sub-network are chemical substructure similarities, side effect similarities, and Anatomical Therapeutic Chemical (ATC) code similarities. In total, similarities among 5089 drugs are provided by the integration of these resources [21]. The latest versions of data resources used to generate the six matrices are provided according to a detailed description of that dataset presented on the above-mentioned GitHub and DKR sites. 

Unfortunately, all publicly available databases mentioned in Table 1 are specifically for humans. It seems most available data and information related to animal diseases, gene targets, and drugs are only embedded in the publications, and there are no comprehensive datasets or repositories for them. However, lack of access to this data did not negatively impact the current analysis because of the similarity of mastitis disease in humans with other animals; animal models have been used for most human studies. Therefore, to specialize the results for dairy cows, we added three parts of information to our generated datasets:

1. Key genes with a robust bio-signature in response to bovine mastitis, especially in *E. coli* infection:

Pubmed and Google Scholar were searched to find genes identified based on meta-analysis studies to have a robust bio-signature in *E. coli* mastitis, which were added to the disease-gene relation part of the dataset shown in Table 1.

2. Functionally related diseases or biological processes associated with bovine mastitis:

The Pathway Studio web tool 12.0.1.5 was used to construct a network of disease or cell processes that were functionally associated with mastitis or bovine mastitis. Pathway Studio is a pathway analysis tool that incorporates some commercial and public databases such as BIND [23], KEGG, and GO [24], utilizing the ResNet Mammalian database. Moreover, it also uses the powerful text-mining tool MedScan to seek the latest information from PubMed and other public sources (Elsevier-Ariadne Genomics, Rockville, MD) [25]. For increased confidence, only relationships which were reported by two or more references were selected. This information has been added to the disease similarity part of the dataset shown in Table 1.

3. Relevant drugs and antibiotics to *E. coli* mastitis:

With a review of the literature, we were able to develop a comprehensive list of drugs or antibiotics that have been used to treat *E. coli* mastitis. These drugs were added to the drug-disease relation part of the dataset in Table 1.

### 2.2. Running Heter-LP

**Figure 1:**
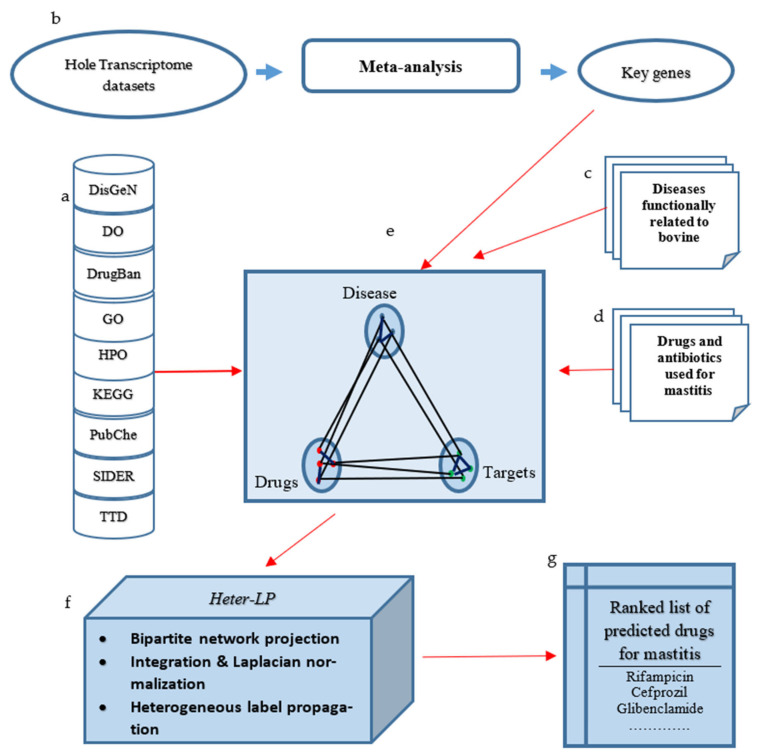
The workflow for this research. (**a**) Data related to diseases, drugs, and their targets gathered from different data sources (Table 1). (**b**) Key genes with robust bio-signatures and key regulatory effects in response to *E. coli* (Table 2). (**c**) Diseases or biological processes functionally related to mastitis identified by using the Pathway Studio web tool (Figure 2). (**d**) Drugs and antibiotics relevant to *E. coli* mastitis gathered by literature mining (Table 3). (**e**) A suitable heterogeneous network constructed by integration of data from parts A, B, C, D (**f**) Running the Heter-LP algorithm on the constructed network to predict important relations involved in mastitis (described in Section 2.2). (**g**) Predicted drugs, ranked according to their score computed by Heter-LP (Table 4 and Supplementary Table S2).

After constructing the datasets as described in the previous sections, they were introduced into the Heter-LP code via six matrices. Heter-LP was implemented in C#; its pseudo code and the workflow are presented in [22], and it is available through GitHub and the DKR website (links above). The Heter-LP output is a ranked list of predicted important links related to *E. coli* mastitis, which were not identified in the input data. Predicted links are in descending order sorted according to their potential probability of existence. The workflow is shown in Figure 1. 

## 3. Results

### 3.1. Basic Similarities and Relations 

An updated version of data based on Table 1 resources has been provided on GitHub (https://github.com/MLotfiSH/Heter-LP, accessed on 26 March 2021) and the DKR site (http://dkr.iut.ac.ir/projects, accessed on 28 March 2021).

### 3.2. Disease Genes 

Genes/proteins with a robust bio-signature in response to mastitis, especially in *E. coli* infection, are listed in Table 2.

### 3.3. Disease Similarity Data 

**Figure 2:**
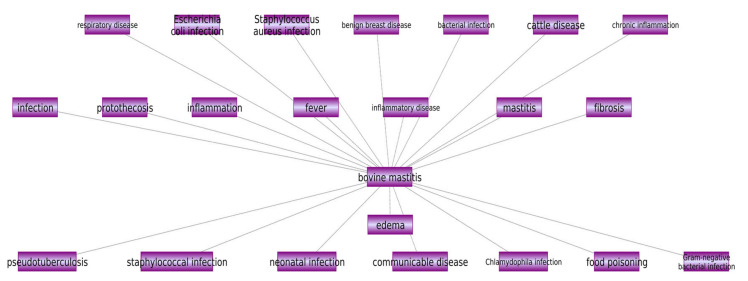
Disease network related to mastitis constructed by using Pathway Studio web tool (based on at least two references).

All relations between mastitis or bovine mastitis and other diseases or cell processes are indicated in Figure 2. Additional details and references are provided in Supplementary Table S1. 

### 3.4. Drugs and Disease 

Drugs or antibiotics that have been used to treat *E. coli* mastitis are listed in Table 3.

As shown, during the current research, we could provide valuable biological information related to *E. coli* mastitis by comprehensive literature mining, including a list of key candidate genes, drugs reported for treatment, and diseases associated with *E. coli* mastitis. Overall, the most important finding of this research is the repositioning of antibiotics or drugs for managing *E. coli* mastitis in dairy cattle. Based on Heter-LP categorization, there are two kinds of predictions, known and novel [22]. The top predicted drugs and antibiotics associated with *E. coli* mastitis are presented in Table 4.

Most of the drugs listed in Table 4 have been reported in the literature as treatments for *E. coli* mastitis (Table 3). These results demonstrate that Heter-LP could correctly identify known relations, indicating that the novel compounds may be realistic predictions. All predicted results of Heter-LP are presented in Supplementary Table S2.

### 3.5. Discussion

While the pharmaceutical industry has explored drug repositioning to identify novel treatments for diseases, this work has been hampered by the lack of a fundamental and systematic approach. Machine learning-based pattern discovery has opened a new vista in early mastitis detection [37,38,39,40] as well as drug repurposing [41,42]. This research used the biological algorithm, Heter-LP, to reposition antibiotics for managing *E. coli* mastitis in dairy cattle. The utility of this new algorithm to discover new drug repositioning possibilities for rare diseases in humans has been explored previously [21]. 

Data available in the public repositories and other specialized biological information for *E. coli* mastitis, including crucial genes, antibiotics, or drugs for treatment, and associated disease or cell processes, were used as input data for the Heter-LP algorithm. Based on the results, we have introduced a list of the most likely candidate drugs to be used as therapeutic strategies against *E. coli* infection. It is noteworthy that these drugs have been suggested among more than 11,000 different drugs, which could help to accelerate and facilitate the drug identification process. Indeed, this list of recommended drugs is valuable for pharmaceutical scientists or veterinarians to find a commercial and productive medicine or combination of two or more active compounds. In the following section, we have tried to validate and confirm most of these new predictions by review of available scientific literature.

Among the list presented in Table 4, Penicillin G (also known as Benzylpenicillin), Rifampicin, Cefprozil, and Cefadroxil are antibiotics. Recent research has shown that Rifampicin could be used as a solo medical therapy in humans for chronic mastitis [43]. Cefprozil, a second-generation cephalosporin antibiotic, is strictly approved worldwide to treat mastitis disease in dairy cattle. Lipopolysaccharides on the outer membrane of Gram-negative bacteria such as *E. coli* are an important barrier protecting against toxic compounds, including antibiotics and hosts’ innate immune molecules such as cationic antimicrobial peptides. These bacteria use a wide variety of mechanisms to resist antimicrobials [44,45]. 

Glibenclamide is an anti-diabetic drug in a class of medications known as sulfonylureas, closely related to sulfonamide antibiotics. Sulfonamides are also occasionally used to treat septicemia caused by coliform mastitis in dairy cattle [46]. It has been shown that the effects of inflammatory markers (TNFα and NFκB), and activation of cell injury or cell death markers (IgG endocytosis and caspase-3), are significantly reduced with glibenclamide treatment [47].

Ipratropium (another new drug listed in Table 4) has been shown to partially protect the lungs against inflammation by reducing neutrophilic infiltration. This protective effect is associated with a reduction in MMP-9 activity, which plays an essential pro-inflammatory role in the acute inflammatory process [48]. 

It has been demonstrated that hypothyroidism with a low level of thyroxine is associated with signs of low-grade inflammation (raised C-reactive protein levels), which may be elicited by a raised triglyceride level or be an independent effect of an intracellular hypometabolic state or a combination of the two [49]. Also, other research has shown that l-Thyroxine treatment of patients with subclinical hypothyroidism can reduce inflammation [50]. As we know, acute inflammation is the main disorder of intramammary *E. coli* infections. Therefore, these drugs, individually or in combination, could be excellent candidates to reduce or treat clinical signs of *E. coli* mastitis. 

Salbutamol, the other predicted drug listed in Table 4, has been shown to decrease acute and chronic inflammation by regulating inflammation mediators, including decreasing myeloperoxidase (MPO) activity and lipid peroxidation (LPO) and increasing the activity of superoxide dismutase (SOD) and level of glutathione (GSH) during the acute phase of inflammation, possibly through the stimulation of β-2 adrenergic receptors [51].

Carbidopa has been used as a treatment for Parkinson’s disease. New research has demonstrated that it inhibits early events in T-cell activation and promotes the development of anti-inflammatory effects. Thus, it has been suggested as a potential therapeutic for the management and/or treatment of inflammatory and autoimmune disorders in humans [52].

## 4. Conclusions

Integration of different levels of systems biology data, including drug, disease, and gene target information, using the Heter-LP algorithm enabled us to introduce novel drugs relevant to *E. coli* mastitis. Based on these results, it can be concluded that we could successfully predict drugs/compounds that can be used as suitable alternatives for the treatment of *E. coli* mastitis using the Heter-LP algorithm. Predicted relationships can be used by pharmaceutical scientists or veterinarians to find commercially efficacious medicines or a combination of two or more active compounds to treat this infectious disease.

## Figures and Tables

**Table 1:** Resources of data related to each sub-network and the number of nodes in each one.

Sub-Network	Using Criterion	Resource	Number of Nodes	
			In Each Resource	In Total
Drugs	Chemical substructure similarities	PubChem ^1^	1103	5089
	Side effect similarities	SIDER ^2^	888	
	Anatomical Therapeutic Chemical (ATC) code similarities	KEGG ^3^	4867	
Diseases	Disease-gene similarities	DisGeNET ^4^	3295	9886
	Similarities based on ICD-10 classification ^5^	KEGG	1366	
	Semantic similarities based on Disease Ontology (DO) ^7^	DOSE package in R ^6^	6560	
	Semantic similarities based on GO ^9^	GOSemSim package in R ^8^	1550	
Targets	Semantic similarities based on HPO ^10^	HPOSim package in R ^11^	979	2940
	Semantic similarities based on DO	DOSE package in R	1092	
	Similarities based on KEGG	KEGG	1132	
Drug-disease	__	Therapeutic Target Database (TTD) ^12^	Drugs: 6931	Drugs: 7382
			Diseases: 1418	
		KEGG	Drugs: 1052	Diseases: 1970
			Diseases: 592	
Drug-target	__	DrugBank ^13^	Drugs: 1521	Drugs: 3350
			Targets: 1346	
		KEGG	Drugs: 2440	Targets: 1415
			Targets: 335	
Disease-target	__	DisGeNET	Diseases: 577	Diseases: 1838
			Targets: 2403	
		KEGG	Diseases: 1271	Targets: 4066
			Targets: 2563	

^1^https://pubchem.ncbi.nlm.nih.gov/score_matrix/score_matrix.cgi, accessed on 3 January 2021; ^2^ http://sideeffects.embl.de/, accessed on 4 January 2021; ^3^ Kyoto Encyclopedia of Genes and Genomes (http://www.kegg.jp, accessed on 1 January 2021); ^4^ http://www.disgenet.org, accessed on 1 January 2021; ^5^ International Statistical Classification of Diseases and Related Health Problems-10, (https://apps.who.int/iris/handle/10665/246208, accessed on 9 January 2021); ^6^ Disease Ontology Semantic and Enrichment analysis (https://bioconductor.org/packages/release/bioc/html/DOSE.html, accessed on 8 January 2021); ^7^ http://disease-ontology.org/, accessed on 4 January 2021; ^8^ https://bioconductor.org/packages/release/bioc/html/GOSemSim.htm, accessed on 9 January 2021; ^9^ Gene Ontology (http://www.geneontology.org/, accessed on 9 January 2021; ^10^ Human Phenotype Ontology, https://hpo.jax.org/app/, accessed on 3 January 2021; ^11^ https://mran.microsoft.com/snapshot/2014-10-20/web/packages/HPOSim/index.html, accessed on 6 January 2021; ^12^ http://bidd.nus.edu.sg/group/cjttd/, accessed on 7 January 2021; ^13^ http://drugbank.ca, accessed on 8 January 2021.

**Table 2:** The key genes or regulators with robust bio-signatures in response to *E. coli* mastitis reported in previous meta-analysis-based transcriptome studies.

Mastitis-Associated Genes	Reference	Technique
CXCL2, CXCL8, GRO1, CFB, ZC3H12A, CCL20, NFKBIZ, S100A9, S100A8, PDE4B, CASP4, HP	[14]	meta-analysis of microarray data
MAPK1, TP53 (p53), SP1, MAPK14, INS, EGF, AKT1, IFNG, MAPK3, MAPK8, VEGFA, MMP2, BCL2, IL10	[26]	meta-analysis of microarray data
MMP9, IL18, GAPDH, CXCL8, IL6, IL1B, TLR2, GRO1, ICAM1, VCAM1, CXCL2, CCL20, CXCL6, IL8RB, IL1A, CCL3, CCL2, NFKBIA, IL1RN, TIMP1	[27]	integration of three microarray datasets
BCL2,BNBD-9-LIKE, BOLA-RDA, C1S, C2,C3, C4BPA, C6, CCDC80, CCL20, CCL3, CCL4, CCL5, CCR5, CD14, CFB, CMTM8, COL17A1, COL1A2, COTL1, CRISPLD2, CXCL11, CXCL16, CYBA, DEFB10, DEFB4A, EGFLAM, FCER1G, FGL1, FGR, FMOD, FN1, HAPLN1, HMOX1, IL1A, IL1B, ITGB6, KERA, KIT, LAP, LBP, LOC504773, LOXL1, LOXL4, LPL, LPO, LTF, LUM, LYZ2, MFAP4, MFGE8, MSR1, MSTN, MYOC, NCF1, NFKBIZ, NOS2, NTN4, OGN, OLR1, ORM1, POSTN, PRELP, PRSS2, PTAFR, PTX3, PYCARD, RAB27A, RSAD2, S100A12, SAA3, SELP, SERPINA3-1, SERPINF1, SERPINF2, SRGN, TAP1, TFF3, TGFB2, THBS1, TLR2, VEGFC, VLDLR, VNN1	[28]	meta-analysis of microarray data

**Table 3:** List of known drugs reported in literature to treat *E. coli* mastitis.

Row	Drug or Antibiotic	Reference
1	Ampicillin	[19]
2	Aspirin	[29]
3	Ceftazidime	[19]
4	Cephalexin	[19]
5	Cephapirin (Cefoperazone, Ceftiofur, Cefquinome)	[18]
6	Chloramphenicol	[30]
7	Cinoxacin	[31]
8	Ciprofloxacin	[19,31]
9	Dexamethasone	[31]
10	DHS (dihydrostreptomycin sesquisulfate sa)	[19]
11	Flunixin meglumine	[32]
12	Fluoroquinolones (enrofloxacin, danofloxacin, marbofloxacin)	[18]
13	Gentamicin	[19,30]
14	Isoflupredone acetate	[29]
15	Ketoprofen	[19]
16	Meloxicam	[33]
17	Oxytetracycline	[34]
18	Penethamate hydriodide	[33]
19	Polymixin	[35]
20	Prednisolone	[36]
21	Tetracycline	[19]
22	Trimethoprim	[19]
23	Sulfadoxine	[34]
24	Sulfamethoxazole	[30]
25	Sulfadiazine	[19]

**Table 4:** Thirty top predicted drugs associated with *E. coli* mastitis by the Heter-LP algorithm.

Row	Drug	Ranking Score	Verification
1	Cefoperazone	0.005000691	Known drug
2	Meloxicam	0.004998696	Known drug
3	Cephapirin	0.003363298	Known drug
4	Cephalexin	0.003362269	Known drug
5	Oxytetracycline	0.003352667	Known drug
6	Cinoxacin	0.003351841	Known drug
7	Ketoprofen	0.003350183	Known drug
8	Aspirin	0.002526886	Known drug
9	Ampicillin	0.001301824	Known drug
10	Ceftazidime	0.001164398	Known drug
11	Tetracycline	0.001162658	Known drug
12	Chloramphenicol	0.000958009	Known drug
13	Gentamicin	0.000937666	Known drug
14	Ciprofloxacin	0.000680685	Known drug
15	Dexamethasone	0.000618516	Known drug
16	Prednisolone	0.000513524	Known drug
17	Penicillin G	8.63 × 10^−5^	New drug
18	Leucovorin	8.19 × 10^−5^	New drug
19	Rifampicin	7.91 × 10^−5^	New drug
20	Cefprozil	7.87 × 10^−5^	New drug
21	Ipratropium	7.81 × 10^−5^	New drug
22	Cefadroxil	7.77 × 10^−5^	New drug
23	Clidinium	7.66 × 10^−5^	New drug
24	Lopinavir	7.64 × 10^−5^	New drug
25	Glibenclamide	7.61 × 10^−5^	New drug
26	Thyroxine	7.57 × 10^−5^	New drug
27	Salbutamol	7.55 × 10^−5^	New drug
28	Carbidopa	7.51 × 10^−5^	New drug
29	Benzquinamide	7.50 × 10^−5^	New drug
30	Diethylpropion	7.49 × 10^−5^	New drug

## Data Availability

The database presented in this study is available in https://github.com/MLotfiSH/Heter-LP, http://dkr.iut.ac.ir/projects.

## Supplementary Materials

The following are available online at https://www.mdpi.com/article/10.3390/ani12010029/s1, Supplementary Table S1. Known diseases related to mastitis or bovine mastitis. Supplementary Table S2. Predicted drug repositioning associated with *E. coli* mastitis by Heter-LP algorithm
